# Duck hepatitis A virus utilizes PCBP2 to facilitate viral translation and replication

**DOI:** 10.1186/s13567-024-01369-9

**Published:** 2024-09-19

**Authors:** Chenxia Xu, Yurui Jiang, Mingshu Wang, Anchun Cheng, Wei Zhang, Xumin Ou, Di Sun, Qiao Yang, Ying Wu, Bin Tian, Yu He, Zhen Wu, Shaqiu Zhang, Xinxin Zhao, Juan Huang, Dekang Zhu, Shun Chen, Mafeng Liu, Renyong Jia

**Affiliations:** 1 Institute of Veterinary Medicine and Immunology, Sichuan Agricultural University, Chengdu, 611130 China; 2 Key Laboratory of Animal Disease and Human Health of Sichuan Province, Chengdu, 611130 China; 3 International Joint Research Center for Animal Disease Prevention and Control of Sichuan Province, Chengdu, 611130 China; 4 Engineering Research Center of Southwest Animal Disease Prevention and Control Technology, Ministry of Education of the People’s Republic of China, Chengdu, 611130 China; 5 Research Center of Avian Disease, College of Veterinary Medicine, Sichuan Agricultural University, Chengdu, 611130 China; 6 Sinopharm Yangzhou VAC Biological Engineering Co., Ltd., Yangzhou, 225100 China

**Keywords:** DHAV-1, PCBP2, IRES, 3D^pol^, translation, replication

## Abstract

Duck hepatitis A virus type 1 (DHAV-1) is an important member of the *Picornaviridae* family that causes highly fatal hepatitis in ducklings. Since picornaviruses have small genomes with limited coding capacity, they must utilize host proteins for viral cap-independent translation and RNA replication. Here, we report the role of duck poly(rC)-binding protein 2 (PCBP2) in regulating the replication and translation of DHAV-1. During DHAV-1 infection, PCBP2 expression was upregulated. A biotinylated RNA pull-down assay revealed that PCBP2 positively regulates DHAV-1 translation through specific interactions with structural domains II and III of the DHAV-1 internal ribosome entry site (IRES). Further studies revealed that PCBP2 promotes DHAV-1 replication via an interaction of its KH1 domain (aa 1–92) with DHAV-1 3D^pol^. Thus, our studies demonstrated the specific role of PCBP2 in regulating DHAV-1 translation and replication, revealing a novel mechanism by which host‒virus interactions regulate viral translation and replication. These findings contribute to further understanding of the pathogenesis of picornavirus infections.

## Introduction

Duck hepatitis A virus (DHAV), which belongs to the *Avihepatovirus* genus in the *Picornaviridae* family, mainly infects 1- to 4-week-old ducklings, causing neurological symptoms such as opisthotonos, spasms, and convulsions [1, 2]. Clinically, DHAV can be classified as DHAV-1, DHAV-2 [3], or DHAV-3 [4]. Among them, DHAV-1 is the most common and is widespread worldwide, seriously jeopardizing the duck industry. DHAV-1 features a single-stranded, positive-sense RNA genome approximately 7.7 kb in length that consists of an open reading frame (ORF), a highly structured 5′ untranslated region (5′ UTR), and a 3′ untranslated region (3′ UTR) with a poly(A) tail [5]. The viral genome encodes a 252-kDa polyprotein that is processed into one structural region (P1) and two nonstructural regions (P2 and P3), which are further cleaved into precursors and mature proteins (VP1 to VP4, 2A to 2C, and 3A to 3D) [5–7]. Among the mature proteins, the DHAV-1 3D protein functions as a viral RNA-dependent RNA polymerase (RdRp) and plays a major role in viral genome replication [8].

Studies have shown that the 5′ UTR of the picornavirus genome harbors an internal ribosome entry site (IRES) for translation initiation [9–11]. Currently, at least five different types of IRESs have been identified in picornaviruses, and each type is characterized by a distinct secondary structure and eukaryotic initiation factor/internal ribosome entry site trans-acting factor (eIF/ITAF) requirements [11, 12]. Viruses with genomes containing type I IRES elements include poliovirus (PV), coxsackievirus (CVB), and rhinovirus (RV) [13–15]; those with genomes containing type II IRES elements include foot and mouth disease virus (FMDV) and encephalomyocarditis virus (EMCV) [16]. The hepatitis A virus (HAV) genome contains a type III IRES [17, 18]. Type IV IRESs were discovered in flaviviruses and later reported in picornaviruses, specifically in Senecavirus A (SVA) [9]. Type V IRESs are found in Aichi virus A in the *Picornaviridae* family, and for some viruses, effective translation initiation requires the DExH-box protein DHX29 [19, 20]. Structural models of all IRES types have been proposed, but only a few structures have been experimentally determined. Significant differences exist among IRESs on the basis of RNA secondary structure and RNA-binding protein requirements. Like that of other picornaviruses, the 5′ UTR of DHAV-1 contains an IRES that shares features with type IV picornavirus IRES elements [21]. The structural core of the type IV HCV and classical swine fever virus (CSFV) IRESs comprises the domain II and domain III regions [22, 23]. Although it contains structural domains similar to those of the type IV picornavirus IRES, the DHAV IRES element is clearly distinct from the type IV IRES in that it possesses two extra domains, Id and Ie, which are not found in any other type IV IRES [21]. The exact mechanism of IRES-mediated translation initiation has not been elucidated; however, it has been postulated that the interaction of trans-acting host factors with cis-acting stem‒loop structures and helices results in the recruitment of translation factors and/or the stabilization of RNA for translation [24]. To date, the only cellular protein identified to play a definitive role in both the RNA replication and IRES-mediated translation of picornaviruses is poly(rC)-binding protein (PCBP) [25–27].

PCBP (also known as hnRNP E and αCP) is a cellular RNA-binding protein involved in the stabilization and translational control of specific cellular mRNAs [28–31]. There are four isoforms of PCBPs, each having three hnRNP K-homology domains (KH domains), termed KH1, KH2, and KH3, which are consensus RNA-binding domains that fold according to a β_1_α_1_α_2_β_2_β_3_α_3_ motif [32–36]. These proteins bind RNAs through their KH domains and exhibit specificity for polypyrimidine tracts on target RNAs [37]. Studies have shown that during PV infection, PCBP2 binds to stem‒loop IV of the PV IRES (a type I IRES) to form a ribonucleoprotein (RNP) complex required for the synthesis of viral polyproteins [26, 38]. In addition, PCBP2 binds to stem‒loop I (a cloverleaf structure) of the PV 5′ UTR with the viral protease 3CD to form a ternary complex that is necessary to initiate negative-strand RNA synthesis [25, 39, 40]. Interestingly, PCBP2 is cleaved at the linker between the KH2 and KH3 domains during the mid-to-late phase of PV infection [41]. Rushika Perera et al. proposed that through the loss of the KH3 domain and therefore the loss of its ability to function in translation, PCBP2 can mediate the switch from viral translation to RNA replication [41]. Therefore, PCBP2 is recognized as a key protein that mediates the switch from viral translation to RNA replication. However, the DHAV-1 5′ UTR does not have a cloverleaf structure, and the IRES is a unique type IV IRES; therefore, whether DHAV-1 utilizes the host protein PCBP2 to regulate its life cycle is unknown.

## Materials and methods

### Cell, strain, and antibody

Duck embryo fibroblasts (DEFs) were extracted from 9–11-day-old duck embryos (specific pathogen-free) and cultured in Dulbecco’s modified Eagle’s medium (DMEM, Gibco) supplemented with 10% newborn calf serum (NBS, Gibco). The DHAV-1 H strain (GenBank accession number: JQ301467) and the engineered *Escherichia coli* DH5α bacterium used in this study were provided by the Poultry Disease Research Center of Sichuan Agricultural University. A mouse anti-Flag monoclonal antibody (Cat: M185-3 S) and a mouse anti-HA monoclonal antibody (Cat: M132-3) were purchased from Medical & Biological Laboratories Co., Ltd. A rabbit anti-HA monoclonal antibody (Cat: AF2305), a mouse IgG antibody (Cat: A7028) and an HRP-conjugated goat anti-mouse IgG (Cat: A0216) were purchased from Beyotime Co., Ltd. A rabbit anti-PCBP2 polyclonal antibody (Cat: A2531) and an HRP-conjugated goat anti-mouse IgG heavy chain antibody (Cat: AS064) were purchased from ABclonal Technology Co., Ltd. A rabbit anti-VP3 antibody was prepared in our laboratory [42]. A rabbit anti-beta (β)-actin antibody (Cat: 20536-1-AP) was obtained from Proteintech Co., Ltd. An Alexa Fluor™ 568-conjugated goat anti-mouse IgG antibody (Cat: A11004) and an Alexa Fluor™ 488-conjugated goat anti-rabbit IgG antibody (Cat: A11008) were purchased from Thermo Fisher Scientific Co., Ltd.

### Plasmids

The eukaryotic expression plasmids pCAGGS-3D-HA, the pCAGGS vector, and the pET28a (+) vector were preserved and provided by the Poultry Disease Research Center of Sichuan Agricultural University. The 3 × Flag-tagged PCBP2, 3 × Flag-tagged truncated PCBP2 (KH1, KH2, KH3, ΔKH1, ΔKH2 and ΔKH3) and 6 × His-tagged plasmids used in this study were constructed as follows. The corresponding cDNAs were amplified by RT-PCR using total RNA extracted from DEFs as a template, subcloned and inserted into the pCAGGS vector or pET-28a (+) vector. The primers used in this study were synthesized by Shanghai Bioengineering Co., Ltd., and the primer sequences are shown in Table 1.

**Table 1:** **All sequences of primers used in this experiment**

Primer name	Sequence (5′-3′)	Refs.
pCAGGS-3 × Flag-PCBP2	GATGACGACGATAAGCTCGAGATGGACACCGGCGTCATCGA	New
	TTGGCAGAGGGAAAAAGATCTTCAGTAGGGAGAGAACCTCT	New
pCAGGS-3 × Flag-KH1	GATGACGACGATAAGCTCGAGATGGACACCGGCGTCATCGA	New
	TTGGCAGAGGGAAAA AGATCTTTACGTGCTGTTGGTCATGGAGC	New
pCAGGS-3 × Flag-KH2	GATGACGACGATAAGCTCGAGGCCACC ATGATCAGCAGCTCCATGACCAA	New
	TTGGCAGAGGGAAAAAGATCTTTACGCATCCAAACCTGCACTGA	New
pCAGGS-3 × Flag-KH3	GATGACGACGATAAGCTCGAGGCCACCATGCCCCCGAAGGGCGTCACCAT	New
	TTGGCAGAGGGAAAAAGATCTTCAGTAGGGAGAGAACCTCT	New
pCAGGS-3 × Flag-ΔKH1	GATGACGACGATAAGCTCGAGGCCACCATGGCCGCCAGCCGGCCCCCGGT	New
	TTGGCAGAGGGAAAAAGATCTTCAGTAGGGAGAGAACCTCT	New
pCAGGS-3 × Flag-ΔKH2	ATGACCAACAGCACGCCCCCGAAGGGCGTCACCAT	New
	GACGCCCTTCGGGGGCGTGCTGTTGGTCATGGAGC	New
pCAGGS-3 × Flag-ΔKH3	GATGACGACGATAAGCTCGAGATGGACACCGGCGTCATTGA	New
	TTGGCAGAGGGAAAAAGATCTTTACGCATCCAAACCTGCACTGA	New
pET-28a (+)-PCBP2	CGCGGATCCGAATTCGAGCTCATGGACACCGGCGTCATTGA	New
	GTGGTGGTGGTGGTGCTCGAGGTAGGGAGAGAACCTCTGCT	New
IRES-Id + Ie	TAATACGACTCACTATATTGTGGTGGTTAGCCAACCA	New
	CAGAATGCAATTAATCCT	New
IRES-Ie	TAATACGACTCACTATATCTGGTGTAATGATCCCATG	New
	CAGAATGCAATTAATCCT	New
IRES-II	TAATACGACTCACTATAGGGGAAGGCTAGTCTATGCC	New
	CACACTCACCTACAACCT	New
IRES-III	TAATACGACTCACTATAGGGTGGTCTAGAGTAGGCAC	New
	AGTCTACTGGTATTATAG	New
PCBP2	GGGCGCAGATCAAAATTGCC	New
	AGGAGGGTTTAGCGCTTTCT	New
β-actin	TACGCCAACACGGTGCTG	
	GATTCATCATACTCCTGCTTG	

“New” refers to primers designed and synthesized in this paper.

### Expression and purification of the PCBP2 protein

After the constructed plasmid pET-28a (+)-PCBP2 was transformed into *Escherichia coli* BL21(DE3), protein expression was induced by the addition of 0.8 mmol/L isopropyl-β-d-thiogalactopyranoside (IPTG) at 25 °C for 8 h. Recombinant PCBP2-6 × His protein was purified with Ni–NTA agarose resin (Sigma). Protein purity was determined by 12% sodium dodecyl sulfate (SDS)-polyacrylamide gel electrophoresis (PAGE), and protein concentration was determined using a TaKaRa BCA protein assay kit.

### PCBP2 knockdown

siRNAs targeting the PCBP2 gene and negative control siRNA were synthesized by GenePharma. When the cells reached 80% confluence, they were transfected with siRNAs using Lipofectamine™ 2000 according to the manufacturer's instructions.

### RNA extraction and qPCR

Total RNA was extracted from the samples using RNAiso Plus Reagent (TaKaRa) according to the manufacturer’s instructions. Viral copies were determined according to the one-step TaqMan fluorescent quantitative RT-PCR method constructed in our laboratory [43, 44]. For cytokine detection, total RNA was reverse transcribed into cDNA using the PrimeScript™ RT reagent Kit (Perfect Real Time) (TaKaRa, Japan). PCBP2 and β-actin transcript levels were quantified through quantitative real-time PCR (RT-qPCR) using a SYBR® Premix Ex Taq™ II (Tli RNaseH Plus) Kit (Takara). The relative mRNA expression levels were analysed using the 2 ^−^^ΔΔCt^ method and compared with those of the blank control group. All the data and images were analysed and produced using GraphPad Prism software version 8.

### Virus titration

The degree of virus infectivity was determined by endpoint dilution. Serially diluted samples were used to infect the indicated cells in 48-well plates, and the TCID_50_ was calculated using the Reed–Muench method.

### Western blotting analysis

The supernatant was discarded from the transfected cells, and the cells were lysed with radioimmunoprecipitation assay (RIPA) buffer (Beyotime) containing 1% PMSF (Beyotime). The proteins were separated by SDS-PAGE and subsequently transferred to PVDF membranes (Bio-Rad). The PVDF membranes were blocked with 5% nonfat milk powder at 37 °C for 3 h and incubated overnight (4 °C) with mouse anti-Flag (1:5000), mouse anti-HA (1:5000), rabbit anti-HA (1:5000), rabbit anti-VP3 (1:800), rabbit anti-PCBP2 (1:1000), and rabbit anti-β-actin (1:5000) primary antibodies. HRP-labelled goat anti-rabbit IgG (1:5000) or HRP-labelled goat anti-mouse IgG (1:5000) was used as the secondary antibody and was incubated with the blots for 1 h at 37 °C. The protein bands were visualized via an enhanced chemiluminescence (ECL) (Bio-Rad) detection reagent.

### Coimmunoprecipitation experiment

After 36 h of transfection, the cells were lysed with immunoprecipitation assay lysis buffer (Beyotime Biotechnology), placed on ice for 30 min and then centrifuged at 12 000 × *g* for 10 min at 4 °C. The lysate supernatant was divided into two parts, and mouse anti-Flag or mouse anti-HA monoclonal antibodies were added at a ratio of 1:100 and incubated at 4 °C for 12 h. Then, BeyoMag™ Protein A + G magnetic beads (Beyotime Biotechnology) were added at a ratio of 1:10 and incubated at 37 °C for 1 h. The immune mixtures were washed with PBS to prevent nonspecific binding. Finally, the supernatant was discarded, and 50 µL of PBS was added, followed by the addition of 10 µL of 5 × loading buffer; the mixture was boiled and subjected to western blotting [45, 46].

### Indirect immunofluorescence assay

The cells were transfected according to the appropriate experimental groups, washed three times with PBS 24 h after transfection, fixed with 4% paraformaldehyde at 4 °C overnight, permeabilized with 0.25% Triton X-100 at 4 °C for 30 min, and blocked with 5% bovine serum albumin (BSA) at 37 °C for 2 h. Rabbit anti-HA (1:1000) and mouse anti-Flag (1:1000) antibodies were used as primary antibodies and incubated overnight at 4 °C, followed by incubation with the secondary antibodies Alexa Fluor™568 goat anti-mouse IgG (1:1000) and Alexa Fluor™ 488 goat anti-rabbit IgG (1:1000) at room temperature for 1 h. Finally, the nuclei were labelled with DAPI (1:1000; D9542, Sigma) at room temperature for 15 min and observed under an inverted fluorescence microscope [47].

### Dual-luciferase reporter assay

DEFs in which PCBP2 was knocked down or overexpressed were transfected with CMV-Rluc-IRES-Fluc or CMV-Rluc-Fluc. Samples were collected 24 h after transfection, and firefly and Renilla luciferase activities were analysed via a TransDetect® Double-Luciferase Reporter Assay Kit (TransGen Biotech). The ratio of FLuc expression to RLuc expression represents the relative DHAV-1-IRES activity.

### RNA–protein coimmunoprecipitation

DHAV-1 for 12 h. The cells were washed in PBS, lysed with RIPA buffer (Beyotime Biotechnology) for 30 min and centrifuged at 12 000 × *g* at 4 °C for 10 min to remove cell at a ratio of 1:100 and incubated at 4 ℃ for 12 h. Subsequently, BeyoMag™ Protein A + G (Beyotime Biotechnology) was added to each sample at a ratio of 1:25, and the mixtures were incubated at 4 °C for 4 h. The RNA–protein complexes were washed three times with DEPC water, and total RNA was extracted via RNAiso Plus Reagent (TaKaRa) and reverse transcribed into cDNA via the PrimeScript RT Reagent Kit with gDNA Eraser (TaKaRa). Finally, PCR analysis was performed by using specific primers (forward primer 5ʹ-CCGGAATTCAGCGTCGTTACACTTGACCTCT-3ʹ and reverse primer 5ʹ-CGCGGATCCTTGGTAAGAGTATCCATTTC-3ʹ) for the DHAV-1 IRES, which were subsequently imaged via agarose gel electrophoresis.

### Biotinylated RNA pulldown assay

For the biotinylated RNA pulldown assay, we used the Pierce™ RNA 3'End Desthiobiotinylation Kit (Cat: 20163) (Thermo Fisher Scientific Co., Ltd.) and Pierce™ Magnetic RNA–Protein Pull-Down Kit (Cat: 20164) (Thermo Fisher Scientific Co., Ltd.) according to the manufacturers’ instructions. First, cDNA containing the DHAV-1 IRES (or its truncated region) and the T7 promoter was amplified with the primers shown in Table 1, transcribed in vitro via a TranscriptAid T7 High Yield Transcription Kit (Cat: K0441) (Thermo Fisher Scientific) and purified. The resulting RNA was subsequently labelled with biotin via the Pierce™ RNA 3ʹEnd Desthiobiotinylation Kit (Cat: 20163) and stored at −80 °C. Nonbiotinylated RNA was used in this assay as a control. Subsequently, according to the instructions of the Pierce™ Magnetic RNA–Protein Pull-Down Kit (Cat: 20164), biotin-labelled RNA was first incubated with streptavidin magnetic beads at room temperature for 30 min. Then, 30 µL of the purified PCBP2-6 × His protein or lysates of the transfected DEFs were added, and the RNA-bead complex was incubated at 4 °C for 1 h. Finally, the magnetic beads were washed three times with wash buffer and eluted with 50 µL of elution buffer. The captured proteins were analysed by western blotting [46].

### Statistical analysis

In this study, all the data were analysed via Student’s *t* test or two-way analysis of variance (ANOVA) with GraphPad Prism software version 8, and *P < 0.05, ** P < 0.01, *** P < 0.001, and **** P < 0.0001 were regarded as statistically significant. An “ns” *P* > 0.05 was considered not significant.

## Results

### DHAV-1 induces the expression of PCBP2

**Figure 1:**
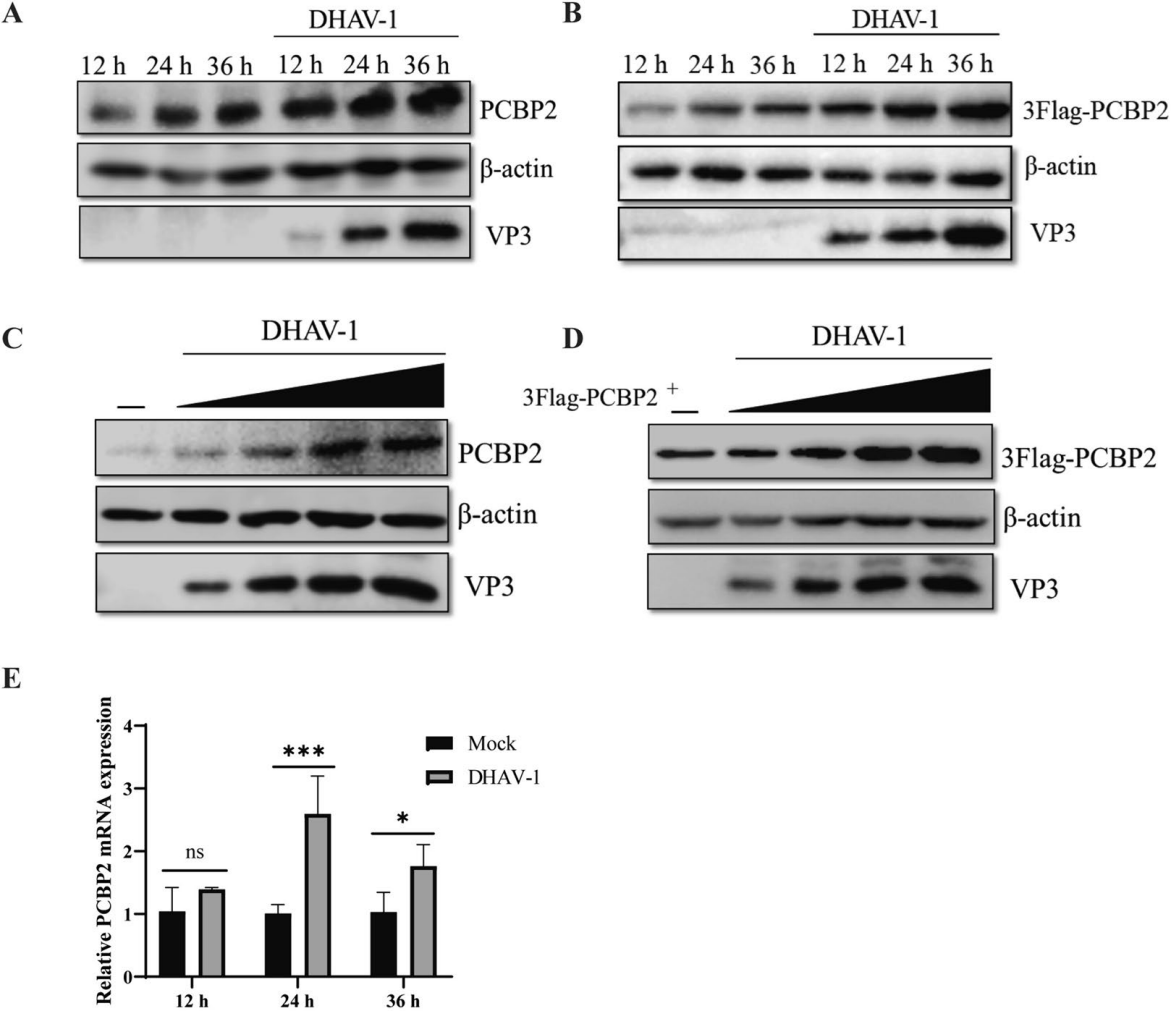
**PCBP2 expression is promoted during DHAV-1 infection.** **A** DEFs were infected with DHAV-1 at an MOI of 0.5 for 12, 24 or 36 h, and the expression of the endogenous PCBP2 protein was measured by western blotting. **B** The pCAGGS-3 × Flag-PCBP2 plasmid was transfected into DEFs for 12 h, after which the cells were infected with DHAV-1 at an MOI of 0.5 for various durations. The expression of 3 × Flag-PCBP2 was measured by western blotting. **C** DEFs were infected with DHAV-1 (MOI = 0.1, 0.5, 1 or 2.5) for 24 h, and the expression of the endogenous PCBP2 protein was measured by western blotting. **D** The pCAGGS-3 × Flag-PCBP2 plasmid was transfected into DEFs for 12 h, after which the cells were infected with DHAV-1 (MOI = 0.1, 0.5, 1 or 2.5) for 24 h. The expression of 3 × Flag-PCBP2 was measured by western blotting. **E** DEFs were infected with DHAV-1 at an MOI of 0.5 for 12, 24 or 36 h, and the cells were collected to measure the mRNA expression of PCBP2 by quantitative RT‒PCR. Differences between two groups were analysed using Student’s *t* test and were considered significant at * P < 0.05, ** P < 0.01, *** P < 0.001, and **** P < 0.0001.

Previous studies have shown that the host protein PCBP2 acts as a switch protein to regulate the replication and translation of PV [41]. However, the role of the PCBP2 protein in the DHAV-1 lifecycle is unclear. To explore the specific role of the PCBP2 protein during DHAV-1 infection, we first investigated the effect of DHAV-1 on PCBP2 expression. PCBP2 protein expression was upregulated during DHAV-1 infection (Figures 1A and B) and increased with increasing DHAV-1 dose (Figures 1C and D). We subsequently examined the effect of DHAV-1 on the PCBP2 mRNA level. The PCBP2 mRNA level in duck embryo fibroblasts (DEFs) was significantly increased by DHAV-1 infection at the 24 h and 36 h time points, with the most obvious increase occurring at 24 h (Figure 1E).

### Effects of PCBP2 overexpression and knockdown on DHAV-1 replication

**Figure 2:**
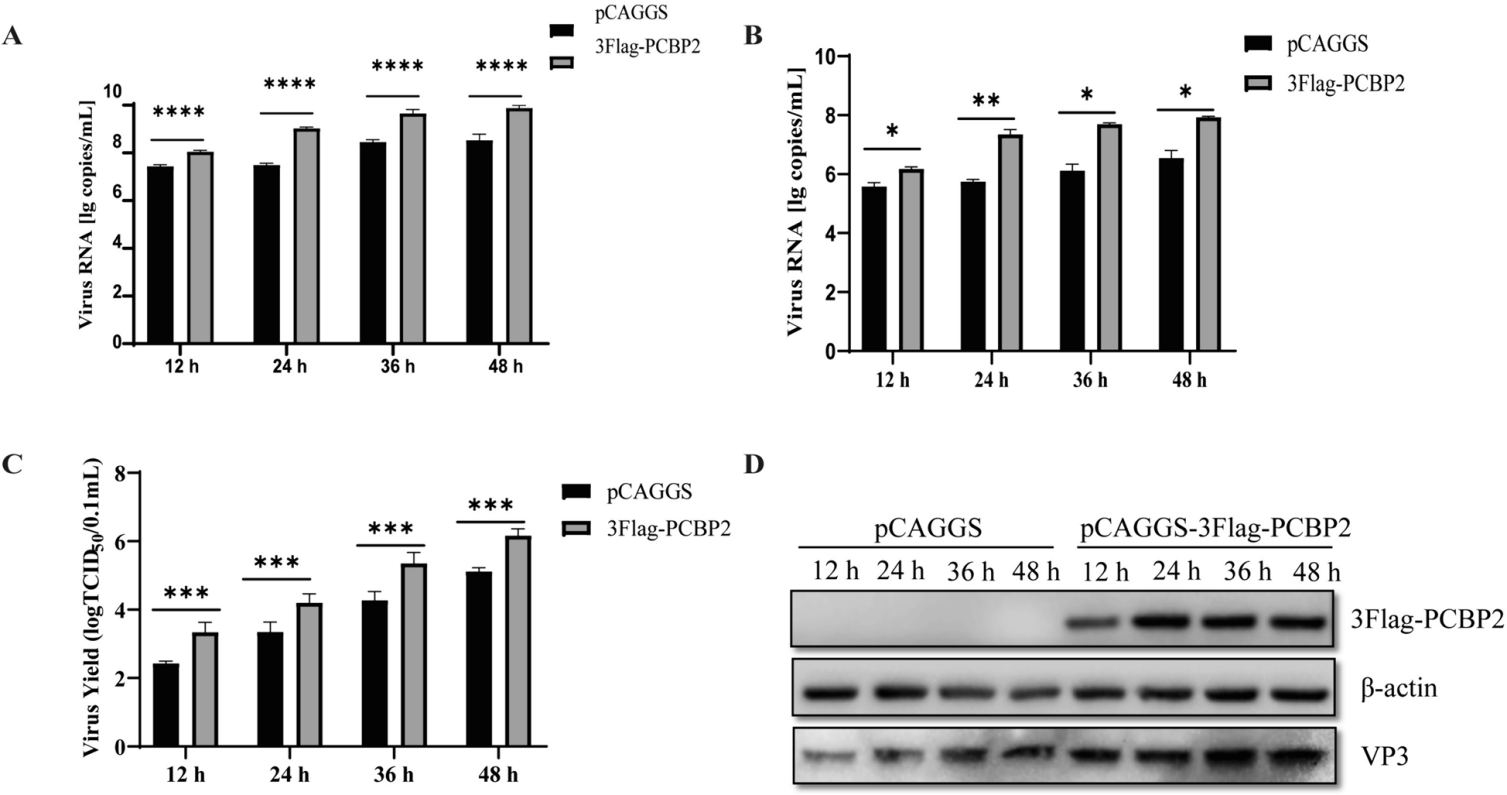
**Overexpression of the PCBP2 protein promotes the replication of DHAV-1.** DEFs were transfected with pCAGGS-3 × Flag-PCBP2 or the pCAGGS empty vector. The transfected cells were then infected with DHAV-1 at an MOI of 0.5, and the virions produced were harvested at 12 h, 24 h, 36 h and 48 h. **A**, **B** Total viral RNA and negative-strand RNA were quantified via one-step TaqMan fluorescent quantitative RT‒PCR. Virus production in the supernatant was analysed by a TCID_50_ assay (**C**), and viral protein expression in the supernatant was analysed by western blotting (**D**). * P < 0.05, ** P < 0.01, *** P < 0.001, **** P < 0.0001.

As PCBP2 expression is regulated by DHAV-1, we sought to determine whether PCBP2 plays a role in DHAV-1 infection. To this end, DEFs were transfected with pCAGGS-3 × Flag-PCBP2 or the empty pCAGGS vector and were then infected with DHAV-1 (MOI = 0.5). The abundances of total DHAV-1 RNA, negative-strand RNA and viral protein were estimated using quantitative RT‒PCR and western blotting. As shown in Figures 2A–C, compared with transfection with the empty vector, overexpression of PCBP2 resulted in significant increases in the production of infectious DHAV-1 progeny throughout the experimental period. After PCBP2 was overexpressed, the level of the DHAV-1 VP3 protein also increased in infected DEFs (Figure 2D).

**Figure 3:**
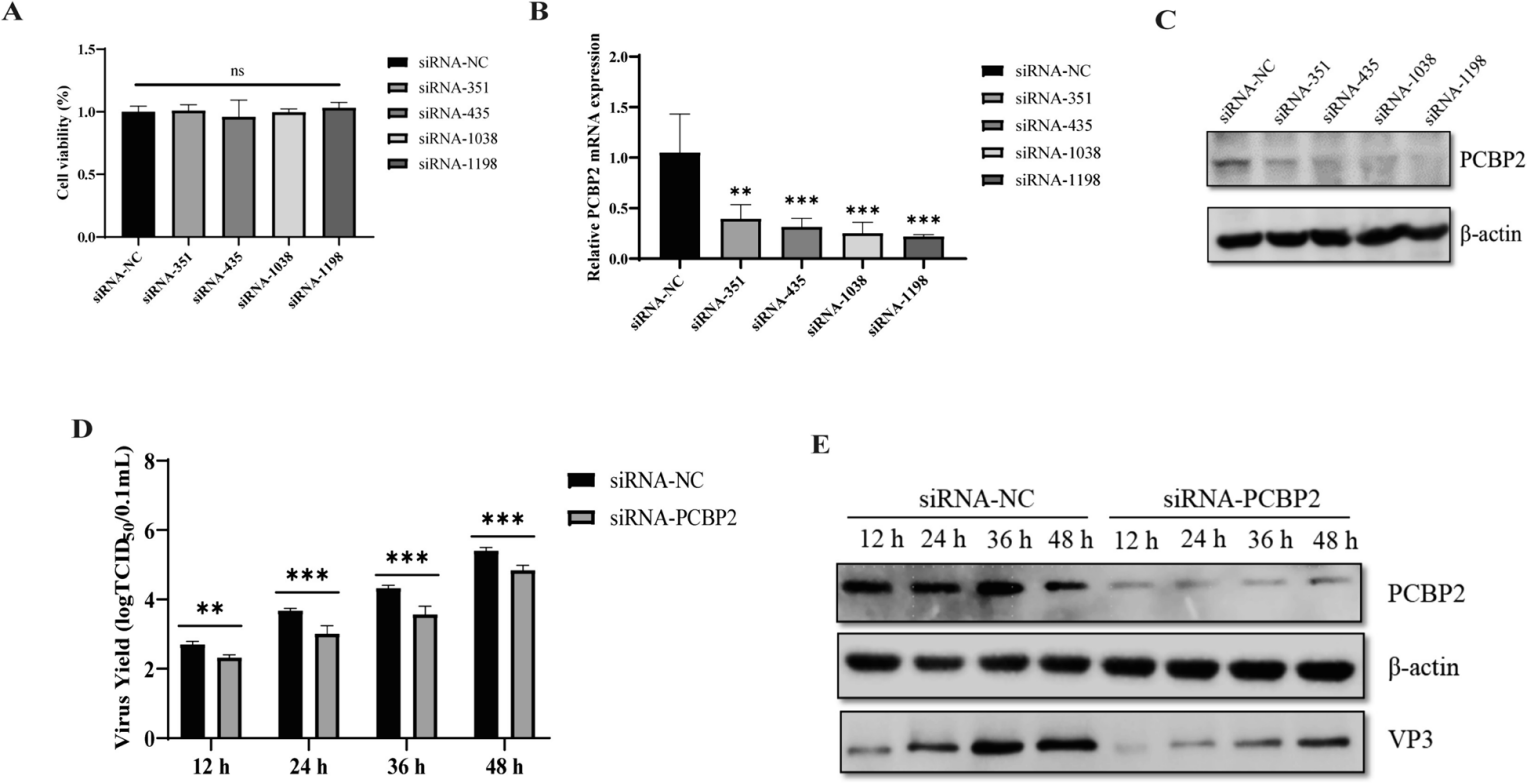
**Knockdown of the PCBP2 protein inhibits replication of DHAV-1.** **A**–**C** DEFs were transfected with siRNA-NC or siRNAs targeting the PCBP2 gene. After 36 h, cell viability was analysed with a CCK-8 assay, and the mRNA expression and protein expression of PCBP2 were analysed via quantitative RT‒PCR and western blotting, respectively. **D**, **E** DEFs were transfected with siRNA-NC or siRNA-PCBP2. After transfection, the cells were infected with DHAV-1 at an MOI of 0.5 and harvested at 12 h, 24 h, 36 h, and 48 h. Viral production in the supernatant was analysed by a TCID_50_ assay (**D**), and viral protein expression in the supernatant was analysed by western blotting (**E**). * P < 0.05, ** P < 0.01, *** P < 0.001, **** P < 0.0001.

The hypothesis that PCBP2 promotes DHAV-1 infection was further verified in DEFs by PCBP2 knockdown using small interfering RNAs (siRNAs). Four specific siRNAs designed to target the PCBP2 coding region were transfected into DEFs, and the effects of the siRNAs on the viability of the DEFs were evaluated using a Cell Counting Kit-8 (CCK8) assay. siRNA transfection did not affect cell viability (Figure 3A) but significantly inhibited the mRNA and protein expression of PCBP2 (Figures 3B and C). siRNA-1198 was then used to evaluate the effect of PCBP2 knockdown on DHAV-1 replication. As shown in Figure 3D, compared with the siRNA control cells, the PCBP2-knockdown cells exhibited inhibited DHAV-1 replication. In addition, PCBP2 knockdown significantly inhibited the expression of the viral VP3 protein (Figure 3E). These results are consistent with the effects of PCBP2 overexpression, indicating that PCBP2 positively regulates DHAV-1 replication.

### PCBP2 interacts with the IRES and regulates DHAV-1 translation

**Figure 4:**
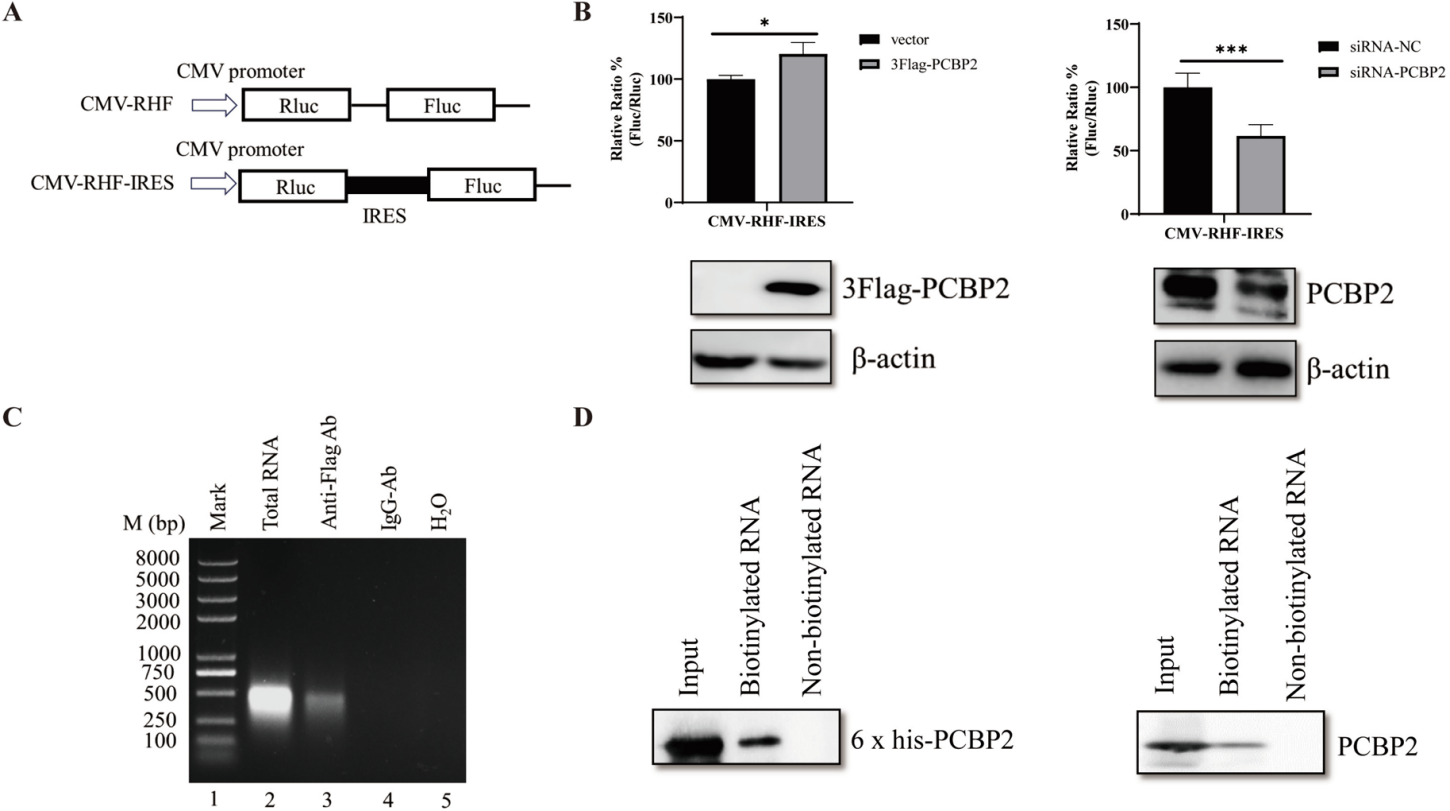
**PCBP2 regulates DHAV-1 translation through interaction with the IRES.** **A** Schematic diagrams of the bicistronic reporter plasmids CMV-RHF and CMV-RHF-IRES. **B** The effects of PCBP2 on DHAV-1 IRES activity were determined via bicistronic reporter plasmids. DEFs in which PCBP2 was overexpressed or knocked down were transfected with the CMV-RHF or CMV-RHF-IRES plasmid. Twenty-four hours post-transfection, the activities of the FLuc and RLuc reporters were measured. The bars in the histogram indicate FLuc/RLuc activity as percentages. The experiments were performed in triplicate, and the results are presented in the bar graph. The expression levels of PCBP2, 3 × Flag-PCBP2, and actin were measured by western blotting. * P < 0.05, ** P < 0.01, *** P < 0.001, **** P < 0.0001. **C** DEFs were transfected with 3 × Flag-PCBP2 and were then infected with DHAV-1 at 12 h post-transfection. The cell lysates were collected, and a mouse anti-HA antibody or mouse IgG was added. The pulled down RNAs were extracted and amplified via PCR using DHAV-1 IRES primers. **D** DHAV-1 IRES RNA was pulled down with purified PCBP2 or from cell lysates transfected with the 3 × Flag-PCBP2 plasmid. The 6 × His-tagged PCBP2 protein was purified by Ni affinity chromatography and then used for pulldown assays. Purified PCBP2 or cell lysates were incubated with the biotinylated or nonbiotinylated IRES, RNA-binding proteins were collected, and their expression was analysed via western blotting.

PCBP2 has also been reported to influence viral translation. Thus, we hypothesized that PCBP2 promotes DHAV-1 replication by affecting IRES-dependent translation. We next examined the effects of PCBP2 overexpression and knockdown on DHAV-1 IRES activity. Bicistronic reporter plasmids with the CMV promoter were used to evaluate DHAV-1 IRES activity (Figure 4A). PCBP2 overexpression promoted but PCBP2 knockdown inhibited viral translation (Figure 4B), suggesting that PCBP2 promotes the IRES-mediated translation of DHAV-1.

The interactions between viral IRESs and cellular proteins in picornavirus-infected cells are crucial for viral replication and translation. To demonstrate the interaction between PCBP2 and the IRES in DHAV-1-infected cells, DEF lysates collected 12 h after infection with DHAV-1 (MOI = 1) were subjected to immunoprecipitation with an antibody against the Flag tag, with normal mouse IgG used as the negative control. The immunocomplexes were isolated, and the DHAV-1 IRES was amplified by PCR using specific primers. As shown in Figure 4C, a cDNA band of the expected size was detected in the anti-Flag immunoprecipitates but not in the mouse IgG or untreated control immunoprecipitates. A biotinylated RNA pulldown assay was subsequently performed to further confirm the interaction of PCBP2 with the DHAV-1 IRES. In this assay, purified PCBP2 and 3 × Flag-PCBP2 in cell lysates bound to biotinylated RNA but not to nonbiotinylated RNA, indicating that PCBP2 specifically interacts with the DHAV-1 IRES RNA (Figure 4D). Taken together, these data demonstrate that PCBP2 interacts with the DHAV-1 IRES and promotes DHAV-1 translation.

### Interaction regions between the DHAV-1 IRES element and the cellular PCBP2 protein

**Figure 5:**
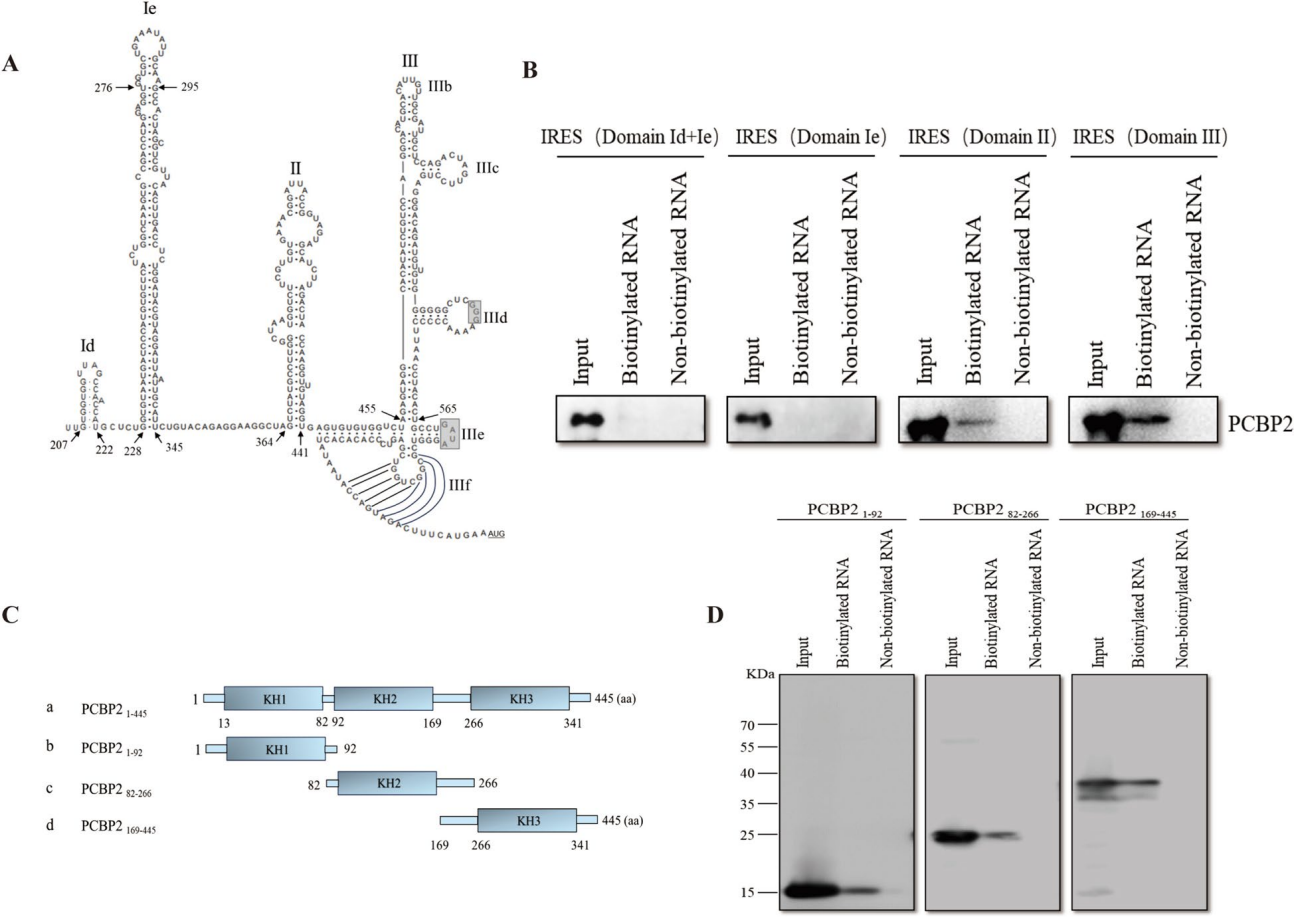
**Identification of interaction regions between the DHAV-1 IRES and PCBP2.** **A** Predicted secondary structure of the DHAV-1 IRES RNA. Four truncations of the IRES were generated: domain Id + Ie, domain Ie, domain II, and domain III. **B** PCBP2 interaction regions in the DHAV-1 IRES. The truncated sequences of the IRES RNA were transcribed in vitro and biotinylated. Purified PCBP2-6 × His was incubated with these biotinylated RNAs. Nonbiotinylated RNA was used in this assay as a control. The RNA–protein complex-bound beads were pulled down and separated by SDS‒PAGE (12%). An anti-PCBP2 antibody was used to detect PCBP2 in the precipitated complexes. **C** Schematic diagram of PCBP2 and its truncation mutants. Three truncation mutants of PCBP2 (a), KH1 (b), KH2 (c) and KH3 (d) were generated and fused with 3 × Flag tags at their N termini. **D** Interaction regions between the PCBP2 protein and the DHAV-1 IRES. Lysates of transfected DEFs were collected 36 h post-transfection and were then incubated with the biotinylated or nonbiotinylated DHAV-1 IRES. Streptavidin beads were used in the pulldown assay, and an anti-PCBP2 antibody was used to detect PCBP2 in the precipitated complexes.

On the basis of the IRES sequence and secondary structure of DHAV-3, we predicted the secondary structure of the DHAV-1 IRES, which was generated by Mfold [48] using the default parameters and visualized with RnaViz 2.0 software [49]. Like the DHAV-3 IRES, the DHAV-1 IRES has two unique domains, Id and Ie, and a structural core consisting of domain II and domain III that is shared by other type IV picornavirus IRES elements [21, 22] (Figure 5A). To identify the IRES domain(s) responsible for binding to PCBP2, four truncations of the IRES—domain Id + Ie, domain Ie, domain II and domain III—were synthesized via in vitro transcription to evaluate their ability to bind PCBP2. As shown in Figure 5B, PCBP2 copurified only with transcripts containing IRES domains II and III, indicating that domain II and domain III of the DHAV-1 IRES are responsible for the binding of PCBP2.

The RNA-binding protein PCBP2 contains three KH domains (Figure 5C) and binds to single-stranded, C-rich RNA sequences [31]. Thus, we constructed PCBP2 truncations fused to a 3 × Flag tag and performed an RNA pulldown assay to identify the KH domain(s) involved in the interaction with the DHAV-1 IRES. By binding the biotinylated DHAV-1 IRES, streptavidin beads captured the IRES-associated truncations KH1, KH2 and KH3 (Figure 5D), indicating that PCBP2 interacts with the DHAV-1 IRES through all three domains, i.e., KH1-3.

### **DHAV-1 3D**^**pol**^** interacts with PCBP2 and promotes PCBP2 protein expression**

**Figure 6:**
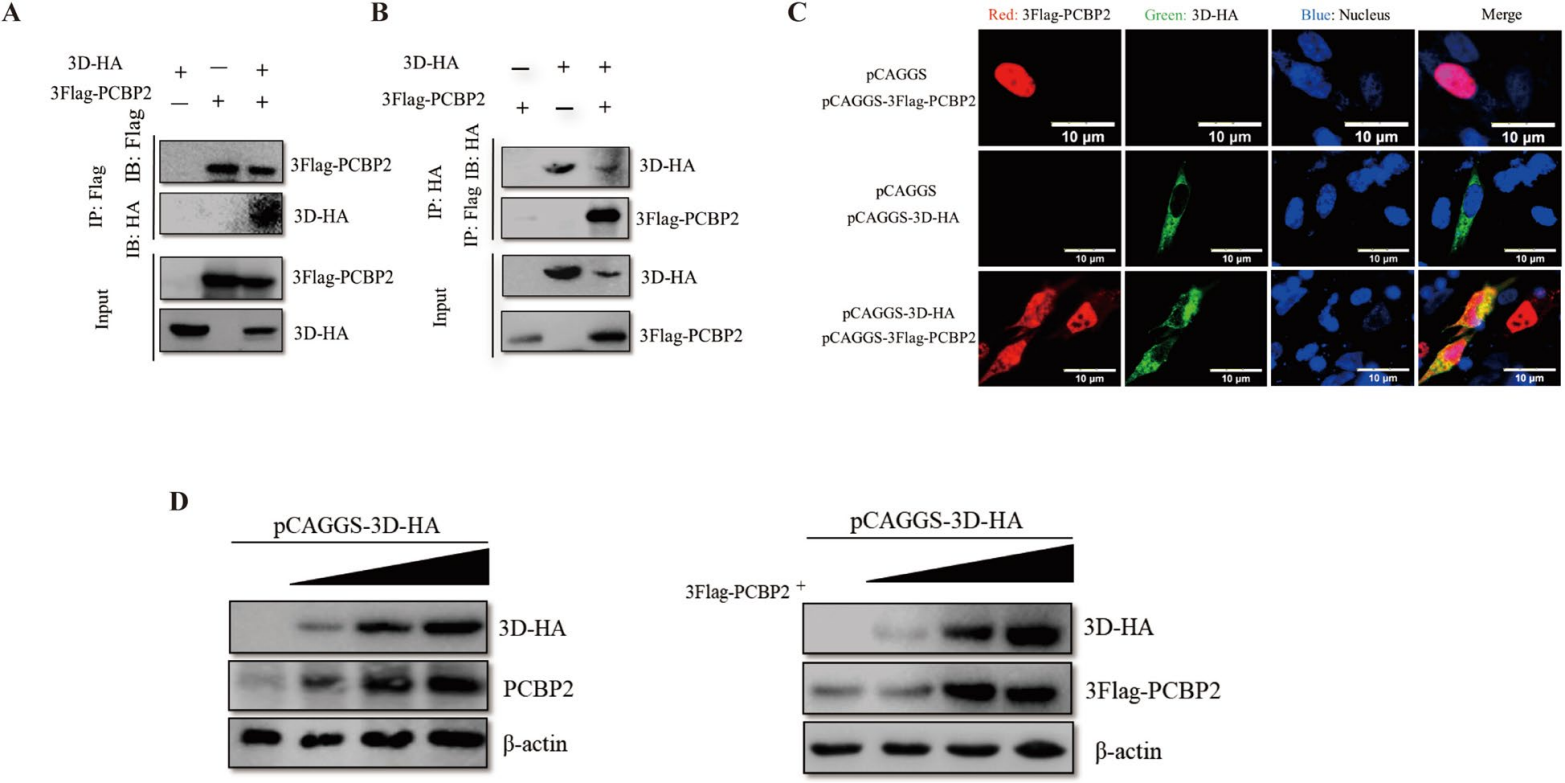
**DHAV-1 3D**^**pol**^** interacts with the PCBP2 protein and promotes its expression.** **A**, **B** DEFs were grown in 6‑well plates, and the cells in each well were transfected with 2 µg of pCAGGS‑3D‑HA or 2 µg of pCAGGS‑3 × Flag-PCBP2 alone or co-transfected with 1 µg of pCAGGS‑3D‑HA and 1 µg of pCAGGS‑3 × Flag-PCBP2. The cells were lysed 36 h after transfection, and the lysates were incubated with a mouse anti-Flag or mouse anti-HA antibody and analysed by immunoblotting with an anti-HA or anti-Flag antibody, respectively. **C** pCAGGS‑3 × Flag‑PCBP2 and pCAGGS‑3D-HA were transfected separately with pCAGGS or pCAGGS‑3 × Flag‑PCBP2 and pCAGGS‑3D-HA were transfected together into cells in 24‑well plates (transfection ratio of 1:1), and cell samples were collected at 24 h after transfection for indirect immunofluorescence experiments to observe the intracellular localization of the 3D^pol^ and PCBP2 proteins. **D** DEFs were grown in 12‑well plates and transfected with different doses of 3D‑HA expression plasmids (the amount transfected into each group was 1 µg, and pCAGGS was used to supplement the insufficient group) or with 0.4 µg of pCAGGS‑3 × Flag-PCBP2 plus different doses of pCAGGS‑3D‑HA (the amount transfected into each group was 1 µg, and pCAGGS was used to supplement the insufficient group). Twenty-four hours after transfection, the cell samples were collected, and the protein expression levels of 3D and PCBP2 were measured.

To further explore the mechanism through which PCBP2 positively regulates DHAV-1 replication, we explored the relationship between PCBP2 and the replication complex. The RdRp 3D polymerase (3D^pol^), an essential component of the viral RNA replication complex, was used as an indicator of viral RNA replication complexes in this study. We first investigated the interaction between PCBP2 and DHAV-1 3D^pol^ via a coimmunoprecipitation assay. A band corresponding to 3D^pol^ or PCBP2 was measured by western blotting when 3 × Flag-PCBP2 or 3D-HA, respectively, was used as the bait protein, indicating that 3D^pol^ can interact with the PCBP2 protein (Figures 6A and 6B). Indirect immunofluorescence also revealed clear colocalization of 3D^pol^ with the PCBP2 protein in the cytoplasm (Figure 6C). To further investigate PCBP2 protein expression, dose‒response assays were subsequently performed, and both the endogenous and exogenous PCBP2 protein levels were found to increase in a dose-dependent manner in response to 3D^pol^ expression (Figure 6D). These results indicate that 3D^pol^ interacts with the PCBP2 protein and promotes its expression.

### **Interaction regions between the DHAV-1 3D**^**pol**^** and host PCBP2 proteins**

**Figure 7:**
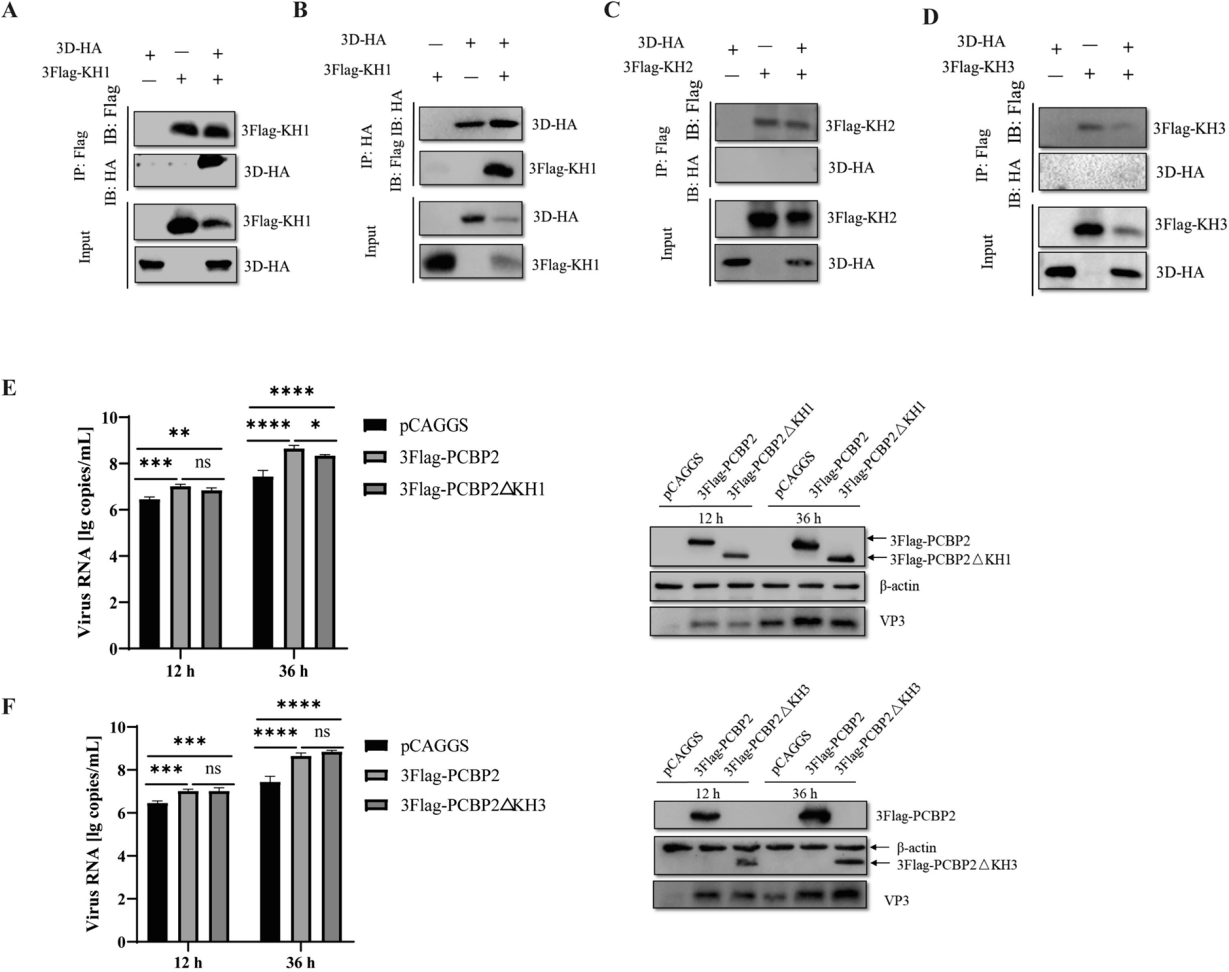
**Interaction regions between the DHAV-1 3D**^**pol**^** and PCBP2 proteins.** **A**–**D** Co-IP assay of exogenous 3 × Flag-tagged truncated PCBP2 and 3D^pol^. The cells were lysed 36 h after transfection, and the lysates were incubated with a mouse anti-Flag or mouse anti-HA antibody and analysed by immunoblotting with an anti-HA or anti-Flag antibody, respectively. **E**, **F** DEFs were transfected with 3 × Flag-PCBP2, 3 × Flag-PCBP2ΔKH1/3 × Flag-PCBP2ΔKH3, or the control pCAGGS vector and were then infected with DHAV-1 at an MOI of 0.5. The viral copy number was quantified via one-step TaqMan fluorescent quantitative RT–PCR. Western blotting was performed to measure the expression of full-length 3 × Flag-PCBP2, 3 × Flag-PCBP2ΔKH1/3 × Flag-PCBP2ΔKH3, and viral VP3 in DEFs. * P < 0.05, ** P < 0.01, *** P < 0.001, and **** P < 0.0001.

Three PCBP2 truncations were utilized to further determine the interaction region(s) in PCBP2 (Figure 5C). The immunoprecipitation results revealed that the PCBP2 truncation aa 1–92 coprecipitated with 3D^pol^ in DEFs (Figure 7A–D). These results indicate that PCBP2 interacts with 3D^pol^ via the KH1 domain. On the basis of the above results, we investigated the effect of the interaction between PCBP2 and 3D^pol^ on DHAV-1 replication. To this end, we explored whether PCBP2 mutants lacking the ability to interact with 3D^pol^ lose the capacity to promote DHAV-1 replication. DEFs overexpressing full-length PCBP2 and the PCBP2Δ1–92 mutant were infected with DHAV-1. The overexpression of both full-length PCBP2 and the PCBP2Δ1–92 mutant promoted the synthesis of viral RNA and the expression of the VP3 protein. However, the promoting effect of the PCBP2Δ1–92 mutant was weaker than that of full-length PCBP2 (Figure 7E), suggesting that the loss of the KH1 domain in PCBP2Δ1–92 attenuated the ability of PCBP2 to promote viral replication. However, PCBP2 mutants with deletions of regions that do not interact with 3D^pol^, such as the KH3 domain, had the same promoting effect on DHAV-1 replication as did full-length PCBP2 (Figure 7F). These results indicate that the interaction of PCBP2 with 3D^pol^ contributes to the promotion of DHAV-1 replication by PCBP2.

## Discussion

Globally, DHAV has been identified in numerous countries in America, Europe, Africa and Asia, and DHAV infection has emerged as a serious hazard that threatens the health of ducklings and results in substantial losses to the duck farming industry. In addition, coinfection of DHAV with other viral pathogens, such as avian influenza virus (AIV), has been observed [50]. Thus, elucidation of the mechanisms underlying viral infection and replication is important for preventing and controlling this disease.

Previous studies have shown that PCBP2 is required for both the translation and replication of some picornavirus genomes, such as those of PV, CVB and HCV. During PV infection, HeLa cell extracts depleted of PCBP2 exhibited ineffective PV RNA translation, but both translation and the production of infectious progeny virions were restored by the addition of recombinant PCBP2 [38]. However, whether DHAV-1 utilizes the host protein PCBP2 for viral translation and replication during infection has not been reported. Therefore, we performed experiments to explore the role of PCBP2 in DHAV-1 translation and replication. In this study, we initially reported that DHAV-1 promotes PCBP2 expression (Figure 1) and demonstrated that PCBP2 promotes DHAV-1 replication (Figures 2 and 3). These findings suggest that DHAV-1 utilizes the host protein PCBP2 to promote viral proliferation.

The typical genome of picornaviruses harbors a well-conserved and highly structured RNA element known as an IRES, which is functionally essential for viral replication and protein translation. Therefore, we further explored the relationship between PCBP2 and the DHAV-1 IRES. A luciferase assay revealed that PCBP2 positively regulated the activity of the IRES (Figure 4B). Subsequently, RNA immunoprecipitation and RNA pulldown assays revealed that PCBP2 can interact with the IRES, indicating that PCBP2 can promote DHAV-1 translation by directly binding to the IRES. Previous reports have shown that in picornaviruses such as PV and CVB3, the host protein PCBP2 binds to the viral IRES and stimulates translation [51, 52], which is consistent with our results. However, unlike those of other picornaviruses, the IRES of DHAV possesses a unique type IV IRES structure [21]. The secondary structure of the predicted DHAV-1 IRES includes core structure domains of type IV IRES elements, domain II and domain III regions, and the additional domains Id and Ie [53–57] (Figure 5A). Our results showed that PCBP2 regulates translation by interacting with domains II and III (Figure 5B). In contrast, for picornaviruses containing type I IRESs, PCBP2 primarily binds to domain IV of the IRES and is essential for translation initiation [25, 26, 58]. However, for type II IRES-containing viruses, PCBP2 is dispensable for translation [27]. Thus, we speculate that differences in the structures of the IRESs of picornaviruses may lead to different roles of PCBP2 in viral infection. Moreover, the additional domains are unique features of the DHAV IRES among type IV IRESs, and the Id is important for DHAV IRES activity [21]. PCBP2 binds domains II and III rather than the unique structure of DHAV-1, suggesting that PCBP2 may play the same role during infections with other type IV IRES viruses.

We further revealed that all three KH domains of PCBP2 bind to the DHAV-1 IRES (Figure 5D), indicating that all three KH domains of PCBP2 are required for DHAV-1 RNA translation and that the combined action of these domains is required for PCBP2 to regulate DHAV-1 translation. However, in viruses such as PV or CVB3, PCBP2 mainly binds to the IRES through the KH3 domain to initiate translation, whereas PCBP2 lacking the KH3 domain cannot function in translation [25, 26, 41, 58, 59]. A comprehensive understanding of the roles of type IV IRESs will contribute to elucidating the replication mechanism and pathogenesis of picornaviruses.

In addition, we examined the association of PCBP2 with viral RNA replication complexes, and 3D^pol^ was used as an indicator of viral RNA replication complexes. Co-IP revealed that PCBP2 coimmunoprecipitated with DHAV-1 3D^pol^ via amino acids 1–92, and this interaction promoted DHAV-1 replication. PCBP2 is cleaved during the mid-to-late phase of PV infection, and the cleaved protein, termed PCBP2-ΔKH3, is unable to function in translation but maintains its activity in viral RNA replication [41]. In enteroviruses, a cloverleaf RNA structure at the 5′ end of the genome functions as a switch to transition from viral translation to replication by interacting with host poly(C)-binding protein 2 (PCBP2) and the viral 3CD^pro^ protein [60]. Thus, scholars have proposed that through the loss of the KH3 domain and therefore the loss of its ability to function in translation, PCBP2 can mediate the switch from viral translation to RNA replication [41]. However, in this study, no proteins cleaved by PCBP2 were detected during DHAV-1 infection, and there was no cloverleaf RNA structure at the 5′ end of DHAV-1. Therefore, we speculate that there is another template-switching mechanism in viruses with type IV IRESs. How DHAV-1 controls the relative levels of viral protein translation and genome replication by modulating the interactions of PCBP2 with viral proteins and the IRES of the genome deserves further study.

Many studies have investigated the polymerase activity of picornavirus 3D^pol^ and its molecular mechanism for catalyzing viral RNA synthesis. However, in addition to its traditional role in replication, 3D^pol^ can interact with several host proteins to benefit viruses in different life cycle stages [61]. Our study identified a host protein that interacts with DHAV-1 3D^pol^, further revealing how viruses regulate and usurp host processes while also helping to elucidate the mechanisms underlying pathogenesis. Several compounds that bind to 3D^pol^ active sites to block viral replication have been reported [62–64]. However, we contend that 3D^pol^ interaction sites with host proteins could serve as novel and promising targets for specific antiviral therapeutics.

In conclusion, our study indicates that PCBP2 is involved in the regulation of DHAV-1 translation by binding to IRES domains II and III and promotes virion production by interacting with 3D^pol^. These results partially elucidate the pathogenesis of DHAV-1 and reveal a novel mechanism by which host‒virus interactions regulate viral translation and replication.

## Data Availability

The data that support the findings of this study are available from the corresponding author, A C, upon reasonable request.
